# Exploring TSLP and IL-33 Serum Levels and Genetic Variants: Unveiling Their Limited Potential as Biomarkers for Mild Asthma in Children

**DOI:** 10.3390/jcm13092542

**Published:** 2024-04-26

**Authors:** Joanna Połomska, Hanna Sikorska-Szaflik, Anna Drabik-Chamerska, Barbara Sozańska, Anna Dębińska

**Affiliations:** Department and Clinic of Paediatrics, Allergology and Cardiology, Wroclaw Medical University, ul. Chałubińskiego 2a, 50-368 Wrocław, Poland; hanna.sikorska-szaflik@umw.edu.pl (H.S.-S.); anna.drabik-chamerska@umw.edu.pl (A.D.-C.); barbara.sozanska@umw.edu.pl (B.S.); anna.debinska@umw.edu.pl (A.D.)

**Keywords:** TSLP, IL-33, polymorphism, asthma, atopy

## Abstract

As the burden of mild asthma is not well understood, the significance of expanding research in the group of patients with mild asthma is emphasized. Thymic stromal lymphopoietin (TSLP) and interleukin 33 (IL-33) are involved in the pathogenesis of atopy and the immune response to inhaled environmental insults, such as allergens, in asthmatic patients. **Objectives**: The objective of this study was to explore the correlation between specific polymorphisms within the genes encoding TSLP and IL-33, as well as the concentrations of TSLP and IL-33 in the serum, and the occurrence of pediatric mild asthma. **Methods**: The analysis encompassed 52 pediatric patients diagnosed with mild bronchial asthma, including both atopic and non-atopic cases, and a control group of 26 non-asthmatic children. Recruitment was conducted through a comprehensive questionnaire. Parameters such as allergic sensitization, serum levels of circulating TSLP and IL-33, and the identification of single-nucleotide polymorphisms in TSLP (rs11466750 and rs2289277) and IL-33 (rs992969 and rs1888909) were assessed for all participants. **Results**: Significantly lower mean serum TSLP concentrations were observed in asthmatic subjects compared to the control group, with atopic asthma patients showing even lower TSLP levels than non-atopic counterparts. No significant differences were found in mean serum IL-33 concentrations between the two groups. Considering the allele model, for both tested SNPs of IL-33, we observed that patients with asthma, atopic asthma, and atopy statistically less frequently possess the risk allele. **Conclusions:** Our study findings suggest that IL-33 and TSLP do not serve as ideal biomarkers for mild asthma in children. Their effectiveness as biomarkers might be more relevant for assessing disease severity rather than identifying asthma in pediatric patients. Further research focusing on the association between TSLP and IL-33 gene polymorphisms and asthma is expected to significantly advance disease management.

## 1. Introduction

While the majority of asthma research has directed its attention toward severe cases [1], it is worth noting that individuals with mild asthma represent the larger portion of the asthma population, ranging from 50 to 75% [2]. As the burden of mild asthma is not well understood, the significance of expanding research in the group of patients with mild asthma is emphasized [3]. Since the definition of mild asthma varies, we have adopted the following criteria: asthma classified at treatment steps 1 or 2, according to the Global Initiative for Asthma (GINA) recommendations, in patients who, one year prior to the examination, were not hospitalized due to asthma exacerbation and currently exhibit good disease control and normal lung function [4].

Understanding the mechanisms underlying the pathogenesis of asthma is crucial for proper patient care. The epithelial cell-derived danger signal mediators interleukin-33 (IL-33) and thymic stromal lymphopoietin (TSLP) have emerged as key players in the intricate network of adaptive Th2 immune responses associated with asthma. In particular, they are a part of the proinflammatory pathway involved in the pathogenesis of bronchial asthma associated with the activation of Th2 cells, myeloid cells and group 2 innate lymphoid cells (ILC2s) that have the ability to create inflammatory responses independently upon mediators produced by T and B lymphocytes [5]. IL-33, a nuclear cytokine, has been implicated in the pathogenesis of allergic diseases as a cytokine abundantly produced by various cell types, including epithelial and endothelial cells. According to current knowledge, IL-33 is released into the airways in a mechanism of passive release as a result of cellular injury and acts as a damage-associated molecular pattern or in a mechanism of active secretion after airway exposure to inhalant allergens without cellular necrosis. It is an essential cytokine in the activation of ILC2, which is an important source of type 2 cytokines [5,6]. TSLP, initially identified as a growth factor for lymphocyte progenitors, is presently acknowledged as a protein predominantly released by epithelial cells (ECs) and epidermal keratinocytes (KCs) in response to irritating stimuli. It is a pleiotropic cytokine that affects the maturation, proliferation, survival and recruitment of multiple cells, such as dendritic cells, T and B cells, and eosinophils, as well as synergistically increases ILC2 proliferation and type 2 cytokine production in combination with their stimulation by IL-33 [7]. Results of published studies have revealed inconsistent associations between circulating cytokines and asthma. It seems that TSLP appears to be critical for the initiation of type 2 inflammatory responses, but its role in sustaining inflammation in the tissue remains not fully understood [8]. Studies that integrate the combined effect of TSLP and IL-33 genotypes in relation to circulating cytokine levels and asthma are lacking. The question is whether asthma risk is modified by the combined effects of TSLP and IL-33 genotypes and whether it is correlated with atopy.

Several single-nucleotide polymorphisms (SNPs) of these cytokines have been reported to be involved in the development of asthma and various other diseases. However, the interaction of the epithelial cell-derived danger signal mediators in regulating the innate allergic response is still not fully elucidated [9]. In the case of IL-33, variations in single nucleotides within this gene can influence the cytokine’s expression and its ability to initiate immune responses. SNPs spanning the IL-33 region mediate the development of asthma among individuals carrying this risk allele [10,11]. Similarly, SNP in the TSLP gene promoter was associated with the expression of this cytokine gene in asthma, increased IgE levels and eosinophilia. Different genetic variants were linked to other type 2 inflammatory diseases, such as atopic dermatitis and chronic rhinosinusitis, potentially influencing the shaping of the immune response in the context of allergic diseases [12,13].

The aim of this study was to analyze the involvement of IL-33 and TSLP in pediatric asthma, exploring their role in children with mild asthma. By unraveling the specific contributions of these cytokines and their SNPs, we aspire to pave the way for a deeper understanding of the immunological pathways involved in pediatric asthma. In the current study, we analyzed the relationship between cytokines and asthma status in children. We evaluated the association between the risk variants of TSLP and IL-33 genes with circulating TSLP and IL-33 levels in the serum of atopic and non-atopic asthmatic children.

## 2. Materials and Methods

### 2.1. Participants

A total of seventy-eight participants of Caucasian ethnicity aged 6–17 at the time of recruitment were enrolled in the study. The study group included fifty-two children (thirty-five boys and seventeen girls) with a diagnosis of mild bronchial asthma, defined as GINA step 1 or step 2, and normal lung function, randomly recruited from patients at the Department of Pediatric Allergology or the Pediatric Allergology Outpatient Clinic (Wroclaw Medical University Hospital, Poland). Questionnaires that included questions regarding sociodemographic status indicators, health status, symptoms of asthma and other allergic diseases, family history of allergic diseases, the number of siblings and contact with an animal in the home environment were completed by all the participants. 

The diagnosis of asthma was established by a physician and based on typical symptoms (wheezing, shortness of breath, cough and chest tightness), clinical history, results of functional lung tests and positive response to a bronchodilator. The control group included twenty-six children (fourteen boys and twelve girls) who met the following criteria: absence of a history of bronchial asthma or any other allergic disease. 

Exclusion criteria were defined as follows: refusal to participate in the study, mental retardation, birth defects of the respiratory system, current allergen immunotherapy or biological treatment in the 3 months preceding the study, autoimmune disorders, current respiratory tract infection or respiratory tract infection in the previous 3 weeks. 

This study was approved by the Ethics Committee of the Wroclaw Medical University, Poland (protocol code 304/2022). All the participants and their legally authorized representatives received information regarding the background and the implementation of the study and they were given the opportunity to withdraw from participation in the study at any time. Written informed consent (including consent for genetic studies) was obtained from children and their guardians before testing. 

All the children enrolled in the study were examined in order to verify the exclusion criteria and perform anthropometric measurements (body weight, height). Allergic sensitization was evaluated by skin prick test (SPT) or serum-specific immunoglobulin E (IgE) measurements for the most common inhalant allergens (grass mix, warty birch pollen, common hazel, alder, cat dander, Dermatophagoides pteronyssinus, Dermatophagoides farinae, Alternaria alternata [HAL Allergy, Leiden, The Netherlands]). Allergic sensitization was defined as the presence of specific IgE (to at least one of the tested allergens) of ≥0.7 kU/L(class II) or the positive result of skin prick tests (induction of a wheal with the largest diameter ≥ 3 mm).

### 2.2. Blood Sample Collection, Storage, and Preparation

The whole blood samples for genetic testing were collected at the time of recruitment. Serum samples from each patient were collected in Eppendorf tubes and stored until the day of determination at −20 °C. Venous blood samples were also collected into tubes containing ethylenediaminetetraacetic acid (EDTA) (Sarstedt AG & Co., Nümbrecht, Germany) for DNA extraction. Samples were stored until the day of determination at −20 °C. The serum levels of TSLP and IL-33 were determined by Human TSLP/thymic stromal lymphopoietin ELISA kit and Human IL-33/interleukin-33 ELISA kit (ELISA; ElAab, Wuhan, China) using the method recommended by the manufacturer. The intensity of staining was read with a Biotek ELX800 plate reader (Biotek, Vinooski, USA) and expressed in pg/mL. Individual samples were measured twice, and the mean values were further analyzed.

### 2.3. Genotyping

Genomic DNA was obtained from EDTA whole blood samples using a QIAamp DNA Blood Mini Kit (Qiagen GmbH, Hilden, Germany). The samples of the 78 subjects were genotyped for specific SNPs, including TSLP rs11466750 and rs2289277, and IL-33 rs992969 and rs1888909. All SNPs were determined with real-time polymerase chain reaction (PCR) assays with subsequent melting curve analysis using SimpleProbe^®^ probes (TibMolbiol, Berlin, Germany), which were complementary to the wild-type sequences. 

PCR was performed at a final volume of 10 µL containing 1 µL of DNA at a concentration of 15–60 ng/µL, 0.5 µL of reagent mix containing specific primers and SimpleProbes^®^ probesat optimized concentration, 0.8 µL of MgCl_2_, and 1 µL of LightCycler^®^FastStart DNA MasterHybProbe (Roche Applied Science, Mannheim, Germany). The reactions were performed on a Light Cycler 1.5 platform (Roche Applied Science, Mannheim, Germany). To test the quality of every step of the genotyping procedures, negative controls for each genotype were included in each reaction.

### 2.4. Statistical Analysis

All statistical analyses were performed with STATISTICA v.13.3 (TIBCO Software Inc., Woy Woy, NSW, Australia) and Excel (Microsoft® Excel®, Microsoft 365 MSO version number 2403, 16.0.17425.20176). The mean value (M), standard deviation (SD), median (Me), lower quartile (Q1) and upper quartile (Q3), as well as the variability range (Min) and (Max), were calculated for all quantitative traits. The assessment of the compatibility of empirical distributions with the theoretical normal distribution was checked by the Shapiro–Wilk test. Qualitative features are shown in the tables in the form of numbers (n) and fractions (%). In all tests, values of *p* < 0.05 were deemed to be statistically significant. The Kruskal–Wallis One-way Analysis of Variance by Ranks was used to compare the results in atopic asthma, non-atopic asthma and the control group. 

The Hardy–Weinberg equilibrium was tested using the χ^2^ goodness-of-fit test to compare the observed genotype frequencies with the expected frequencies among the controls. Differences in genotype frequencies or demographic characteristics between case and control groups were evaluated using the χ^2^ test. The associations of genotypes or alleles with patient groups versus control subjects were determined by computing the odds ratio (OR), 95% confidence interval (95%CI), and *p* values using a logistic regression analysis for crude ORs. To test the dominant model, wild-type homozygotes were compared with heterozygotes and homozygotes for minor alleles. To study a co-dominant model, the three genotype groups were analyzed separately, using wild-type homozygotes as a reference group. In the multiplicative model, genotypes were coded as a 3-level variable for the number of minor alleles.

## 3. Results

### 3.1. Characteristics of Participants

A summary of the characteristics of patients with asthma and the controls is presented in Table 1. The two groups were similar in terms of living conditions (residential area, number of siblings, contact with an animal in the home environment), but the asthmatic group was slightly younger than the control group. There were also differences regarding body mass index (BMI).

Most of the asthmatic children were atopic and had positive personal and family history of atopic diseases (Table 2).

The frequency of occurrence of risk alleles in the examined SNP polymorphisms for TSLP and IL-33 did not differ between the study group and the control group (Table 3).

Characteristics of the study population in relation to the presence of the risk allele are presented in Table 4. 

### 3.2. Results of Measurements

Biological samples were obtained from all 78 children (52 asthmatics and 26 healthy control subjects). We observed a significant difference in the mean serum TSLP concentration, which was lower in asthmatic children than in the control group (Table 5). 

Regarding the mean serum IL-33 concentration, there were no significant differences among the two groups (Table 6).

**Figure 1:**
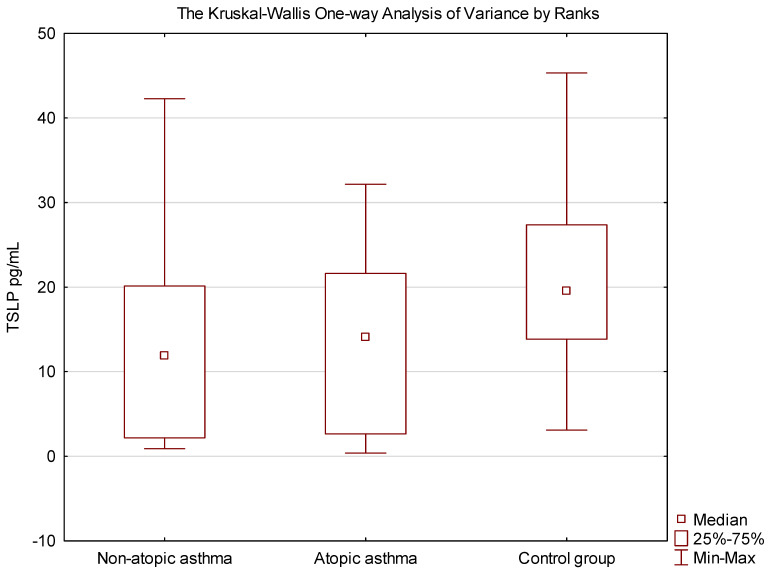
Comparison of the concentrations of TSLP between atopic asthma, non-atopic asthma and controls. The significance of the differences in the assessment of TSLP between asthmatic and controls was verified using the Kruskal–Wallis One-way Analysis of Variance by Ranks (H = 6.988269 *p* = 0.03).

Considering the categorization into groups, patients with atopic asthma exhibited lower concentrations of TSLP than those with non-atopic asthma. Both asthmatics with and without atopy demonstrated lower TSLP levels compared to the control group (Figure 1).

**Figure 2:**
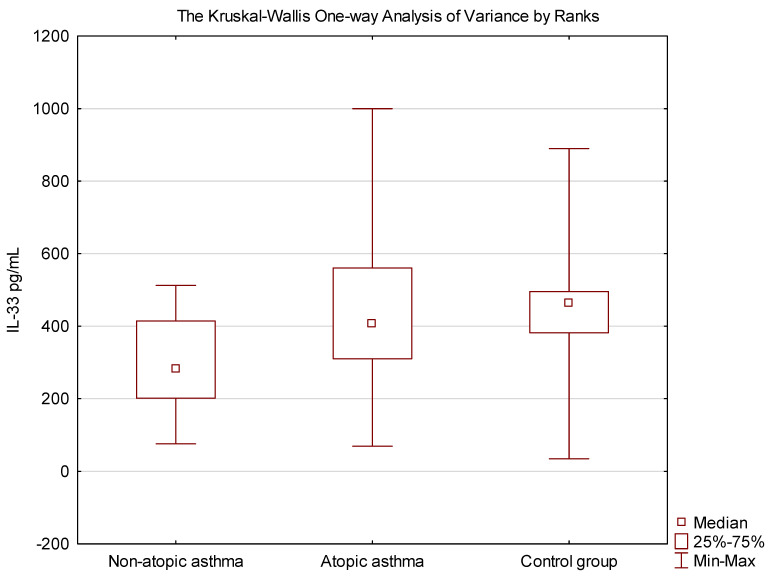
Comparison of the concentrations of IL-33 between atopic asthma, non-atopic asthma and controls. The significance of the differences in the assessment of IL-33 between asthmatic and controls was verified using the Kruskal–Wallis One-way Analysis of Variance by Ranks. (H = 9.216405, *p* = 0.01).

Patients with non-atopic asthma were characterized by a statistically significantly lower concentration of IL-33 compared to the group of patients with atopic asthma (*p* = 0.04) and the control group (*p* = 0.01) (Figure 2).

**Figure 3:**
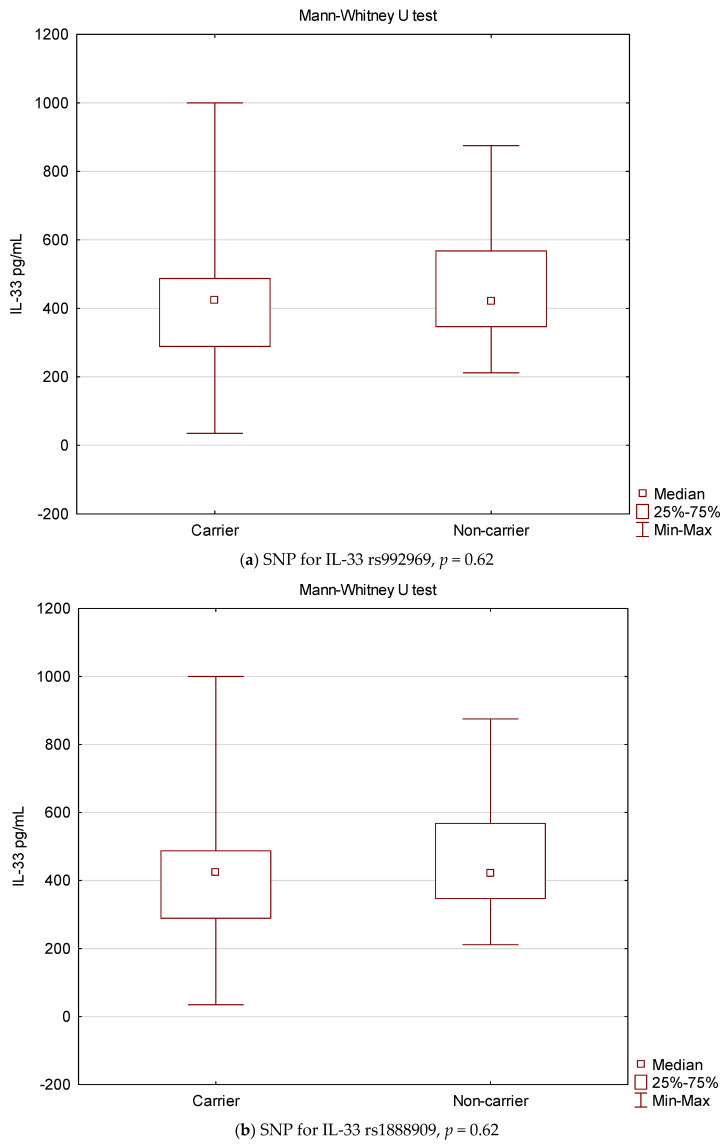

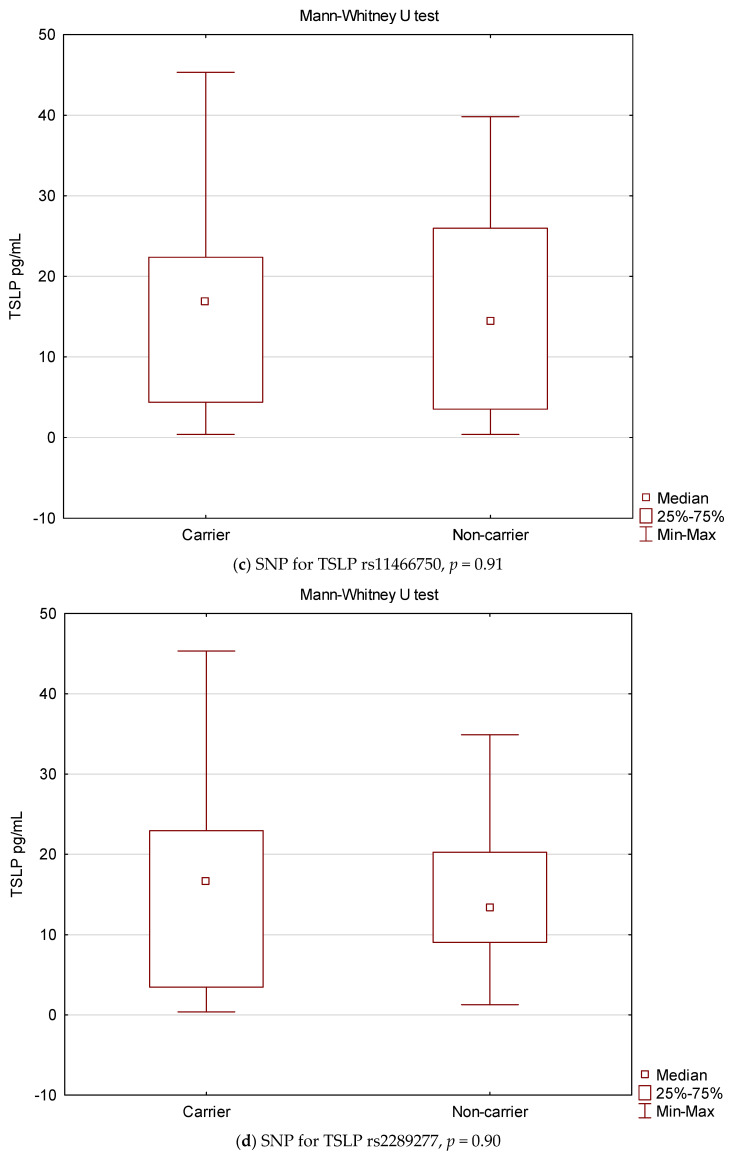
(**a**–**d**) Comparison of concentrations of interleukins between risk variant carriers and wild-type (non-carriers). The significance of the differences in the assessment of TSLP and IL-33 of carriers and non-carriers was verified using the Mann–Whitney U test.

Concentrations of interleukins between risk variant carriers and wild-type (non-carriers) were analyzed to examine the impact of tested genetic variations on IL-33 and TSLP expression at the systemic level. The results are presented in Figure 3a–d.

IL-33 rs992969 SNP, as well as IL-33 rs1888909 SNP, showed a significant association with asthma, atopic asthma and atopy in the allele model. The results of statistical analyses are presented in Table 7. Statistical analyses were also performed for other genetic models for the tested polymorphisms, except for TSLP rs11466750 (no suitable cases, i.e., absence of homozygote individuals in the cases and control groups). Analysis of the IL-33 rs992969 polymorphism revealed a significant association with susceptibility to atopic asthma using the co-dominant model: wild-type genotype versus homozygous genotype for risk allele, *p* = 0.027, RR = 0.49, 95%CI 0.48 ÷ 1.02, as well as using the dominant model: wild-type genotype versus heterozygous and homozygous genotype for risk allele, *p* = 0.035, RR = 0.54, 95%CI 0.53 ÷ 1.08. Similar results were obtained for IL-33 rs992969 polymorphism regarding the association with susceptibility to atopy using the same co-dominant model (*p* = 0.011, RR= 0.08, 95%CI 0.09 ÷ 0.72) and dominant model (*p* = 0.044, RR = 0.12, 95%CI 0.01 ÷ 1.06). Regarding IL-33 rs1888909 polymorphism, a significant association with susceptibility to atopic asthma was revealed in the dominant model (*p* = 0.036, RR = 0.54, 95%CI 0.53 ÷ 1.08). For SNPs in the TSLP gene, there were no significant associations with susceptibility to asthma, atopic asthma or atopy when using three different genetic models tested separately. 

## 4. Discussion

This study investigated the relationship between serum IL-33 and TSLP levels and asthma, along with polymorphisms in the IL-33 and TSLP genes and asthma susceptibility. Prior investigations predominantly documented elevated levels of IL-33 in peripheral blood among asthmatic patients as compared to healthy controls [14]. Studies conducted in children with asthma show similar observations; furthermore, they indicate a correlation between serum IL-33 levels and the severity of the disease—the more severe the symptoms of asthma, the higher the concentration of IL-33 [15,16]. In the present study, no significant difference in IL-33 levels was observed between asthmatic children and healthy controls. A plausible explanation is that children with mild asthma may have low serum levels of IL-33. The findings of our study align with those of another investigation examining the associations between adherence to the Mediterranean diet and serum levels of specific cytokines known to play a pathogenetic role in the airway changes associated with asthma. The researchers measured IL-33 levels in the serum of 44 children with intermittent/mild asthma and 26 healthy controls aged 5–15. The obtained results indicated 313.7 ± 324.2 pg/mL in the case group and 320.5 ± 310.2 pg/mL in the control group, showing no significant elevation of IL-33 levels in asthmatic children compared to healthy controls. Furthermore, the findings of their investigation suggested that adherence to the Mediterranean diet facilitated the production of IL-33. In our study, dietary information regarding the children’s daily intake was not considered [17]. 

Discrepancies in the results of IL-33 concentration measurements across various studies may arise due to the influence of other non-allergic diseases in patients. For example, it has been noted that elevated levels of serum IL-33 and IL-33 receptor genetic polymorphism are evident in patients with multiple sclerosis, suggesting a significant role of this cytokine in the pathogenesis of autoimmune disorders [18]. Participants enrolled in our study had no history of autoimmune diseases that could potentially impact the results. In other published studies, it remains unclear whether participants are free from autoimmune diseases, and the control group is often briefly defined as volunteers without asthma, allergic diseases, or chronic respiratory conditions [18].

In another study, researchers examined the serum levels of IL-33 and TSLP in allergic asthmatics and healthy subjects both before and 24 h after a bronchial allergen challenge [19]. Participants, aged 18–50, were non-smoking individuals with recently diagnosed and untreated asthma and also with positive skin prick test response to the Dermatophagoides pteronyssinus allergen. Healthy, non-atopic volunteers without other chronic respiratory diseases were included as a control group. The bronchial allergen challenge test with inhaled Dermatophagoides pteronyssinus allergen was performed for all participants in the study. Differently from our study, the baseline serum IL-33 level in the allergic asthmatic group was significantly higher, almost twofold, at 65.7 (28.6–119.3) pg/mL, compared to 37.8 (13.0–119.9) pg/mL in healthy subjects. However, the bronchial allergen challenge test did not induce an increase in IL-33 levels in either group, with values of 63.4 (37.4–97.4) pg/mL in asthmatic patients and 38.0 (9.88–95.5) pg/mL in healthy subjects. This indicates the need for further research on the concentration of alarmins, taking into account exposure to the sensitizing allergen, in asthmatic children and adults. 

In an animal model, it was shown that IL-33/ST2-dependent responses of mast cells play a protective rather than a causative role in the development of airway hyper-responsiveness [20]. It appears that there is a necessity for a more detailed characterization of IL-33/ST2-dependent mast cell responses and their relevance in the context of asthma. It is essential to focus on measuring both IL-33 and the soluble form of ST2 rather than solely one of them. We anticipate that our study will contribute to the design of future investigations exploring the role of circulating cytokines in asthma pathogenesis. The results of our study suggest a potential role of other factors capable of modulating the production of key inflammatory mediators in asthma. These factors may impact the production of IL-33 and the secretion of the soluble form of ST2, which acts as an inhibitor of IL-33, preventing the cytokine’s binding to T cells [21].

Studies investigating the role of TSLP in asthma demonstrate a correlation, indicating elevated serum levels of TSLP in asthmatic patients compared to healthy individuals. The authors also discuss associations with male gender, increasing age, and eosinophilia, which are suggested to be linked with higher concentrations of TSLP in the blood [22]. Similarly, in children, higher concentrations of TSLP have been revealed in patients with asthma, but no significant differences were found when dividing the asthmatic group into allergic and non-allergic individuals. Additionally, there was no observed increase in TSLP concentrations during asthma exacerbations [23]. In our study, an unexpected difference in the mean serum TSLP concentration emerged, revealing lower levels in asthmatic children compared to those in the control group. This outcome could be partly attributed to the fact that in our study, participants exhibited mild asthma and maintained normal lung function.

In the previously mentioned study conducted by Kalinauskaite-Zukauske et al., at baseline, the TSLP levels did not differ between the asthma group and the control group (311.7 [216.9–581.7] vs. 319.1 [179.7–435.4] pg/mL). However, a significant change was observed after allergen exposure in the asthmatic group, where the TSLP level increased to 383.5 (261.0–773.3) pg/mL, with no changes observed in healthy controls [19]. In our study, participants were not exposed to allergens; however, exploring the impact of allergen exposure on potential alterations in circulating cytokine concentrations could be of interest. Furthermore, establishing a correlation between the obtained results and specific genetic models warrants investigation.

In our investigation, we assessed the concentration of TSLP in blood serum. However, several studies have been helpful in highlighting the role of assessment of the local concentration of these cytokines in various biological samples derived from lungs. Existing literature suggests that the local expression of TSLP in skin cells at mucosal surfaces might hold greater significance for the disease compared to systemic levels. The latter may not precisely mirror the concentrations present in local tissues [8]. Other researchers suggest that TSLP levels in sputum may be a more reliable predictor of asthma compared to serum TSLP levels [24].

Glück et al. found that IL-33 and TSLP levels in exhaled breath condensate (EBC) were statistically higher in asthma patients compared to controls. Serum IL-33 levels were also elevated in asthma patients, while TSLP showed a non-significant trend. No significant correlations were observed between serum and EBC levels of TSLP and IL-33 in asthma patients, suggesting separate local epithelial and systemic responses [25].

In another study, IL-33 levels were assessed in endobronchial tissue specimens from healthy controls and asthmatic subjects with varying degrees of severity. The results showed that the bronchial epithelium is a significant source of IL-33 in the lung, with elevated expression observed in asthmatic patients. Histological observations suggested that IL-33 release into the airway lumen increases with asthma severity [26]. Fux et al. investigated the role of IL-33 as a mediator of acute allergic response in asthmatic patients and assessed IL-33 levels in bronchoalveolar lavage fluids obtained from segmental allergen challenge. The study revealed the rapid release of IL-33 upon allergen provocation in vivo, with significantly elevated levels observed during the early-phase reaction but not in the late-phase reaction. This suggests dynamic changes in the local concentration of IL-33 in the bronchi. It appears that the concentration of IL-33 may be elevated not only due to cellular necrosis in chronic allergic inflammation but also as a result of an active process of its release [27].

These studies demonstrate the significance of the assessment of circulating IL-33 and TSLP and their local tissue release in the lung to determine their individual and combined effects on asthma. Further research, including measurements of cytokines locally and systemically, as well as investigating the impact of genetic variants, may provide more insights into the pathogenesis of allergic diseases.

The question is whether TSLP can be a cytokine of interest in non-severe asthma as well. TSLP, a cytokine involved in severe asthma treatment, was never previously studied in non-severe asthma. Ibrahim et. al studied plasma TSLP levels in association with asthma characteristics and their evolution among adults with non-severe asthma in a large epidemiological study [28]. They reported for the first time that age, sex, smoking and BMI are determinants of TSLP levels. They also found that high levels of plasma TSLP were associated with the persistence of asthma attacks and dyspnea. Ten-year results suggest that TSLP could be a cytokine of interest in non-severe asthma and non-allergic asthma as a predictive marker of later asthma persistence, suggesting TSLP is a potential biomarker to monitor asthma evolution in adults [28]. This suggests that a cohort study examining changes in the concentration of these alarmins over time while considering the atopic march, as well as periods of asthma exacerbations and remissions, may be of greater value.

In previous studies, associations between various TSLP SNPs and asthma were analyzed [29,30,31]. In our research, we focused on rs2289277 and rs11466750 and did not observe a relationship between the carrier status of any of the studied SNPs and serum TSLP concentration. Comparing asthmatics and non-asthmatics, we did not detect a higher frequency of asthma occurrence in patients carrying the polymorphic risk variant within the investigated SNPs. Murrison et al., while examining the same SNPs and determining their impact on TSLP mRNA expression and TSLP circulating protein levels, concluded that the presence of either of the two investigated SNPs slightly increases the risk of asthma in the studied group of children. Furthermore, they did not observe elevated TSLP levels in carriers but demonstrated increased expression in the upper airways. They emphasized that the occurrence of asthma may be associated not with systemic TSLP secretion but with local secretion [8]. Similarly, Smolinska et al. observed the impact of both TSLP genotype and expression in the nasal epithelium, rather than systemic TSLP, on the increased risk of asthma in children [32].

Examining the TSLP genotype alone may not be sufficient for assessing the risk of developing asthma. Subsequent investigations could gain insights by incorporating TSLP expression alongside the TSLP genotype. 

Similarly, in the case of IL-33, several SNPs have been described that may be associated with the occurrence of asthma [30]. We investigated the rs1888909 and rs992969 genetic variants of IL-33, and among pediatric patients in Poland with one or both polymorphisms, the level of total circulating IL-33 in plasma did not differ significantly between carriers and non-carriers. Furthermore, considering the allele model, we observed that patients with asthma, atopic asthma and atopy statistically less frequently possess the risk allele. This was observed for both tested SNPs of IL-33. Moreover, in the case of IL-33 rs992969 in the dominant and codominant models, a significantly lower frequency of [G] allele was observed in patients with atopic asthma and atopy compared with wild-type homozygotes. A similar trend was observed for IL-33 rs1888909 in patients with atopic asthma. The data we obtained in our study failed to replicate previously published results. The relatively small size of our study population and lack of statistical power are likely to be major problems in achieving an unambiguous outcome, but for now, they are only preliminary.

Analyzing the same SNPs as in our study, Li et al. found that in the European population, carriers of risk alleles at rs1888909 and/or rs992969 exhibit statistically significant IL-33 transcript levels compared to non-carriers of these alleles. Simultaneously, in the Afro-American population, increased IL-33 transcription was observed only in individuals with rs992969. European children possessing the asthma risk allele at either rs1888909 or rs992969 showed higher levels of IL-33 protein in comparison to children who did not carry these alleles [11]. Further investigations revealed the relationship between the rs992969 genotype and eosinophilic asthma, suggesting that having this specific genotype was associated with higher eosinophilia in patients [10].

In another study, the authors focused on the association of the SNP with IL-33 expression in tissues related to asthma. By conducting bronchial epithelial biopsy (BEC) and bronchial alveolar lavage (BAL), they demonstrated higher concentrations of IL-33 in BEC among patients characterized by the presence of the rs992969 SNP. This points to the role of IL-33 as an alarmin and highlights the importance of the SNP we also investigated in the pathogenesis of asthma [33]. Our observations, although ambiguous, may also indicate the pleiotropic effect of the studied cytokines and the complex pathogenesis of asthma in children, with numerous genetic factors associated with the development of this disease.

In addition to the small sample size, other potential limitations of our study should be taken into account. Differences in BMI may well be due to a statistical type I error because of our small selected sample. It would have been perfect if the asthmatic patients and non-asthmatic subjects had been uniform in terms of BMI. In our study, BMI was higher in asthmatic children compared to healthy controls. However, it should be taken into account that this finding is consistent with epidemiological observations indicating higher BMI among asthmatic children [34].

We lack data on specific pollutants and thus cannot examine their individual effects on cytokine levels. Second, it appears that repeated cytokine measurements would potentially offer a more complete picture of immune responses than a single measurement because of better accounting for intrasubject variability or effects from acute exposures [19]. Another weakness is that we had no local cytokine measurements in the airways, thus limiting our assessment of potential effects.

The strength of our study lies in the emphasis on a comprehensive analysis of both cytokines and genetic factors. Another key aspect is the implementation of a uniform study protocol, ensuring consistent measurements across asthmatic children and healthy control pediatric subjects. We hope that our study will contribute to the design of future research, aiming to explore the potential of alarmins as biomarkers in asthma and unravel their role in asthma pathogenesis.

## 5. Conclusions

In conclusion, our findings indicate that in our study, IL-33 and TSLP are not biomarkers of mild asthma in children. Their usefulness as biomarkers may be related to determining disease severity rather than detecting asthma in pediatric patients. We hope that our study will help in the design of further studies to investigate changes in circulating cytokine concentrations in specific genetic models. Further confirmation of our results and experimental studies will be required to better understand the functional implications of these genetic variants in the pathogenesis of asthma and other allergic diseases, which may contribute to the development of effective prevention strategies.

## Figures and Tables

**Table 1:** Characteristics of study participants (*n* = 78).

Characteristic	Study Group *n* = 52	Control Group *n* = 26	*p*
Gender, *n* (%):			0.246
Girls	17 (32.69%)	12 (46.15%)	
Boys	35 (67.31%)	14 (53.85%)	
Age at the time of recruitment (years):			0.019
M ± SD	11.2 ± 3.10	12.69 ± 2.60	
Me [Q1; Q3]	11 [8; 13]	13 [7; 17]	
Min–Max	6–17	7–17	
Age groups, *n* (%):			0.002
6–11 years	31 (59.62%)	6 (23.08%)	
12–17 years	21 (40.38%)	20 (76.92%)	
Residential area:			0.380
Rural	14 (26.92%)	10 (38.46%)	
<15,000 residents	6 (11.54%)	1 (3.85%)	
>15,000 residents	32 (61.54%)	15 (57.69%)	
BMI (kg/m^2^):			0.004
M ± SD	18.30 ± 3.39	12.27 ± 3.47	
Me [Q1; Q3]	17.67 [16.01; 20.44]	20.06 [18.67; 22.83]	
Min–Max	11.90–27.87	14.88–29.65	
Number of siblings:			0.685
M ± SD	1.12 ± 1.10	1.34 ± 1.67	
Me [Q1; Q3]	1 [0; 2]	1 [1; 1]	
Min–Max	0–5	0–9	
Contact with an animal in the home environment:			0.068
Dog	15 (28.85%)	8 (30.77%)	
Cat	14 (26.92%)	10 (38.46%)	

M—mean; SD—standard deviation; Me—median (50%); Q1—lower quartile (25%); Q3—upper quartile (75%); Min—minimum; Max—maximum; *n*—numbers; %—fractions; *p*—*p*-value.

**Table 2:** Asthma and allergy status characteristics in study group (*n* = 52).

Characteristic	Results
Atopy	36/52 (69.23%)
Other atopic diseases of the child	37/52 (71.15%)
Positive family history of atopic diseases	33/52 (63.46%)

**Table 3:** The genotype frequency in study participants (*n* = 78).

Specific SNP	Study Group *n* = 52	Control Group *n* = 26	adj. *p*
TSLP rs11466750			>0.05
AG	16 (30.77%)	14 (53.85%)	
GG	36 (69.23%)	12 (46.15%)	
AA	0 (0.00%)	0 (0.00%)	
TSLP rs2289277			>0.05
CC	19 (36.54%)	6 (23.08%)	
CG	22 (42.31%)	14 (53.85%)	
GG	11 (21.15%)	6 (23.08%)	
IL-33 rs992969			>0.05
AA	7 (13.46%)	0 (0.00%)	
GA	19 (36.54%)	7 (26.92%)	
GG	26 (50.00%)	19 (73.08%)	
IL-33 rs1888909			>0.05
CC	25 (48.07%)	19 (73.08%)	
CT	20 (38.46%)	7 (26.92%)	
TT	7 (13.46%)	0 (0.00%)	

*n*—numbers; %—fractions; adj *p*—adjusted *p*-values used the Bonferroni method.

**Table 4:** The allele distribution in relation to a specific polymorphism.

Specific Single-Nucleotide Polymorphism	Risk Allele	Allele (Frequencies)	
		Study Group	Control Group
TSLP rs11466750	A	G (84.62%) A (15.38%)	G (73.08%) A (26.92%)
TSLP rs2289277	C	G (42.31%) C (57.69%)	G (50.0%) C (50.0%)
IL-33 rs992969	G	A (31.73%) G (68.27%)	A (13.46%) G (86.54%)
IL-33 rs1888909	C	T (32.69%) C (67.31%)	T (13.46%) C (86.54%)

**Table 5:** Serum concentrations of TSLP in participants (*n* = 78).

TSLP (pg/mL)	Study Group *n* = 52	Control Group *n* = 26	*p*
M ± SD	13.35 ±10.94	20.86 ± 10.87	0.008
Me [Q1; Q3]	14.02 [2.63; 20.96]	19.57 [13.84; 27.36]	
Min–Max	0.39–42.29	3.08–45.33	

**Table 6:** Serum concentrations of IL-33 in participants (*n* = 78).

IL-33 (pg/mL)	Study Group *n* = 52	Control Group *n* = 26	*p*
M ± SD	398.99 ± 190.87	448.94 ± 155.45	0.069
Me [Q1; Q3]	384.09 [262.76; 489.99]	463.90 [381.71; 495.21]	
Min–Max	69.33–1000	34.85–889.73	

**Table 7:** Asthma, atopic asthma, non-atopic asthma and atopy risk stratification for analyzed SNPs. The control group comprises all individuals who do not belong to any of the disease groups.

Phenotype	Total *n* (%)	Genotype Status			
		IL-33 rs992969 A vs. G *p*-Value OR (95%CI)	IL-33 rs1888909 T vs. C *p*-Value OR (95%CI)	TSLP rs11466750 G vs. A *p*-Value OR (95%CI)	TSLP rs2289277 G vs. C *p*-Value OR (95%CI)
Asthma	52/78 (66.7%)	*p* = 0.019 0.33 (0.14 ÷ 0.82)	*p* = 0.012 0.32 (0.13 ÷ 0.78)	*p* = 0.090 0.49 (0.22 ÷ 1.11)	*p* = 0.369 1.36 (0.69 ÷ 2.66)
Atopic asthma	36/62 (58.1%)	*p* = 0.021 0.31 (0.12 ÷ 0.79)	*p* = 0.021 0.31 (0.12 ÷ 0.79)	*p* = 0.185 0.54 (0.23 ÷ 1.30)	*p* = 0.717 1.18 (0.58 ÷ 2.41)
Non-atopic asthma	16/42 (38.1%)	*p* = 0.151 0.60 (0.37 ÷ 1.22)	*p* = 0.057 0.56 (0.35 ÷ 1.09)	*p* = 0.172 0.39 (0.11 ÷ 1.30)	*p* = 0.182 1.90 (0.76 ÷ 4.74)
Atopic asthma vs. non-atopic asthma	36/52 (69.2%)	*p* = 0.654 0.93 (0.72 ÷ 1.28)	*p* = 1.000 0.91 (0.37 ÷ 2.22)	*p* = 0.771 1.40 (0.41 ÷ 4.73)	*p* = 0.292 0.62 (0.26 ÷ 1.47)
Atopy/allergic sensitization	36/78 (46.1%)	*p* = 0.045 0.69 (0.50 ÷ 1.02)	*p* = 0.000 0.23 (0.10 ÷ 0.51)	*p* = 0.543 0.73 (0.33 ÷ 1.65)	*p* = 0.872 0.93 (0.49 ÷ 1.75)

## Data Availability

All information of importance for the conclusions is present in the manuscript.
